# Leaders’ ambition and followers’ cheating behavior: The role of performance pressure and leader identification

**DOI:** 10.3389/fpsyg.2023.982328

**Published:** 2023-01-26

**Authors:** Ahmad Adeel, Daisy Mui Hung Kee, Anila Sadaf Mubashir, Sarminah Samad, Yahya Qasim Daghriri

**Affiliations:** ^1^School of Management, Universiti Sains Malaysia (USM), Penang, Malaysia; ^2^Department of Business Education, The University of Chenab, Gujrat, Pakistan; ^3^Department of Management Science, National University of Modern Languages, Islamabad, Pakistan; ^4^Department of Business Administration, College of Business and Administration, Princess Nourah Bint Abdulrahman University, Riyadh, Saudi Arabia; ^5^School of Management, Universiti Sains Malaysia (USM), Penang, Malaysia

**Keywords:** leaders’ ambitions, leader identification, performance pressure, cheating behavior, ambition theory, social identity theory

## Abstract

**Purpose:**

We seek to understand why and how leaders’ actions that are positive from organizational perspectives, drive to engage employees in cheating behaviors.

**Design/methodology/approach:**

The proposed mediated moderation model was tested in two separate studies, study 1 and study 2, with data collected from police officers and employees of Islamic banking respectively, and then analyzed with Mplus for random coefficient models for direct effects, indirect effects, and for mediated moderation.

**Findings:**

It was found that leaders’ ambitions may enhance performance pressure on the subordinates, which in turn promotes their cheating behavior. Overall, we found that the traditional view of ambition theory only emphasizes good mechanisms such as motivation. However, to integrate with a social identity perspective, ambition would also cause pressure and pressure rather than motivation. Additionally, leaders’ ambitions are more strongly and positively related to the performance pressure and cheating behaviors of employees when subordinates also have high leader identification. The findings of this research suggested that leaders’ positive workplace behavior could also spawn subordinates’ unethical behaviors.

**Practical implications:**

Through this research, we can help policymakers understand that leaders’ positive desire in general and ambition, in particular, may not be necessarily associated with subordinates’ positive behaviors. Our results revealed that internalized with performance pressure, the leaders’ ambition is associated with subordinates’ cheating behavior. The findings of this research will help policymakers understand what might be promoting unethical behavior of employees. The cheating behavior of employees is not a singular level phenomenon of subordinates, it could also be triggered by contextual factors. Therefore, in developing policies for reducing the chance of cheating at work, the policymakers should also focus on the contextual factors that might be promoting cheating.

**Originality/value:**

Ambitious leaders tend to demonstrate high performance, also, performance pressure literature focuses efforts of the employees toward high performance. The dark side of these lines of researches is still underexplored. We shifted the conventional focus of understanding to the positive side of ambition and performance pressure by explaining the potential cost in the form of employees’ enhanced cheating behavior. The interplay between the relationship between leaders’ ambition and subordinates’ perception of leader identification also enhanced our understating about the boundary condition of the relationship between leaders’ ambition, performance pressure, and cheating behavior of subordinates.

## Introduction

Cheating behavior refers to unethical acts intended to create unfair advantages or help to attain benefits that an employee would not otherwise be entitled to receive (Shu et al., 2011) is associated with the financial drain of the organizations (Meyer, 2010). Frequently cited examples of cheating behavior include lying to customers, over-reporting, under-reporting, scamming, and deceiving to advance personal interest by creating unfair advantages. The upward trend of cheating has been found in almost every sector from education to manufacturing (McCabe, 2016; Thompson, 2016; Bretag et al., 2018) and from financial to public services (Egan, 2016; Belle and Cantarelli, 2017). Scholars have long recognized cheating as a singular level phenomenon of people (Williamson, 1984; Higgins, 1997) triggered by personal traits (Hilbig and Zettler, 2015). Yet the rise in cheating behavior has made researchers think contextual cues that might affect cheating behavior (Mitchell et al., 2018; Spoelma, 2021).

Scholars have speculated that certain factors within organizations promote employees’ ethical behaviors (Treviño et al., 2014; Mitchell et al., 2018). For instance, leaders’ ethical conduct plays an important role in determining employees’ ethical conduct (Paterson and Huang, 2018). They implement ethical and moral standards that their followers often demonstrate (Krasikova et al., 2013). Their workplace actions “trickles down” to followers at lower levels (Schaubroeck et al., 2012; Adeel et al., 2019a, 2022a); leaders’ positive actions normalize followers unethical intentions (van Gils et al., 2015), and buffers the unethical behavior of employees (Braun and Hornuf, 2018). However, negative actions are translated into followers’ unethical intentions and behaviors (Schyns and Schilling, 2013). While this approach can be useful to explain leaders’ deeds and ethical behavior of subordinates. Scholars are beginning to ask why and how leaders’ actions, which are positive from organizational perspectives, drive to engage employees in unethical behaviors (van Gils et al., 2015).

Our research addresses this issue, taking leaders’ ambition as a desired workplace behavior (Steffens et al., 2018), we develop and test a model that how leaders’ ambition is translated into employees’ cheating behavior. Ambitious leaders seize the opportunities to change things for the betterment (Balda and Mora, 2011), they achieve high goals (McClelland, 1975), which ultimately benefit organizations in the form of high employee commitment and better performance (Steffens et al., 2018). Drawing on ambition theory (Schlesinger, 1966), this research hypothesized leader ambition is a positive workplace action (Steffens et al., 2018) to enhance the cheating behavior of employees. Past research has consistently shown that leaders’ high ambition tend to spawn positive behavior of followers, such as energizing followers for their full potential (Hobfoll, 2011), influencing followers interests (Dilchert, 2007), internalizing followers self-concept (DeRue et al., 2009), and fueling force of betterment (Caro, 2002). In essence, a leader’s ambition would provide all the positive energies that followers need to behave positively at work. However, these studies have ignored the fact that although leaders’ ambition drives followers to engage in positive workplace behaviors, there could be potential risks of being unethical by demonstrating cheating behavior due to the leader’s high ambition.

Followers of an ambitious leader tend to demonstrate high levels of achievements (Dilchert, 2007) or are more result-oriented (Winsborough and Sambath, 2013; Sieberer and Müller, 2017; Nagel et al., 2020), to some extent, these studies support that with high ambitious leader followers have to take more ownership and pressure to achieve leaders’ goals. Thus, when leaders at organizations are driven to succeed, their followers feel more pressure to achieve organizational goals. Based on these arguments, this study uses ambition theory (Schlesinger, 1966) to hypothesize that leaders’ ambition increases performance pressure on employees which in turn enhances chances of employees’ cheating behavior. Social identity theory (Ashforth and Mael, 1989) predicts that employees develop social identification within their organizations when they attribute belongingness to others based on some attraction or the attributes and characteristics they use to define themselves (F. A. Mael and Tetrick, 1992). To reveal boundary condition of the relationship between leader ambition and employees’ performance pressure, in this research, building on social identity theory, we propose and test moderating effect of subordinates’ leader identification on this relationship. To summarizing, we propose a mediated moderation model to systematically analyze how leaders’ ambition is related to employees’ cheating behavior through performance pressure and the boundary conditions thereof. This research will help organizations understand cheating behavior as triggered by ambitions of the leaders thereby developing strategies for normalizing performance pressure and for lowering intentions of employees to cheat at work.

This research intends to make several contributions. We address recent calls to uncover the contextual factors within organizations that enhance employees’ cheating behavior (Treviño et al., 2014; Mitchell et al., 2018). In emerging research on cheating behavior, scholars have speculated that certain organizational factors promote the employees unethical intentions; for instance researcher speculated that several environmental factors contribute to self-interest which may further promote unethical behaviors (Spoelma, 2021; Hillebrandt and Barclay, 2022; Kamran et al., 2022; Malesky et al., 2022). Similarly in the same vein, other researchers found that employees intentions to cheat at work are enhanced when employees need to protect self-interest (Mitchell et al., 2018; Spoelma, 2021). Our study provides unique insight into cheating’s emerging research that how leaders’ positive actions are translated into employees’ cheating behavior. Recent literature has provided evidence that emotions, cognition, and performance goals are translated into cheating intentions (Welsh and Ordóñez, 2014; Mitchell et al., 2018). We shed light on the leadership side that is translating into followers’ cheating behavior. Additionally, the traditional view of ambition only emphasizes the good mechanism such as motivation (Steffens et al., 2018), however, to integrate with the social identity perspective, ambition would also cause pressure, rather than motivation.

Our research also provides unique insights by offering a needed explanation of the antecedents that promote performance pressure. More specifically, the antecedents are not singular in nature and have roots within the organizations. The presumed benefit of performance pressure is to motivate individuals to increase their efforts for higher achievements which ultimately benefit organizations. We also contributed to the list of negative consequences of performance pressure by offering employees’ cheating behavior as affected by both leaders’ ambition and performance pressure. Finally, we investigated leaders’ actions and followers’ responses in a single study, which is rarely investigated in management research (Day, 2014; Bastardoz and Van Vugt, 2019).

## Literature review and hypotheses

### Understanding cheating behavior

Cheating at work is an employee’s unethical act for creating unfair advantages and attaining benefits (Shu et al., 2011). These unfair acts of employees cost billions of dollars to the organizations (GRTB–Global Retail Theft Barometer, 2016), thus, organizations seek to find ways to reduce cheating. Cheating and unethical behaviors were traditionally conceptualized as individual-level psychological phenomena; therefore, the focus of research remained with understating and investigating psychological factors that contribute to employees’ cheating and unethical behavior (Hsiao and Yang, 2011). Employee cheating is a result of cost and benefit calculation (Becker, 1993) that includes three main predictors (i) expected benefits (ii) the possibility and probability of cheating detection (iii) and the cost in the form of punishment and its magnitude in case of cheating detection. The empirical evidence linking these three predictors with cheating differ in their impact (Nagin and Pogarsky, 2003). The behavioral ethical literature concerns with the influence of intervening factors that influence cheating behavior of employees; contextual factors, professional background, cultural context, and the relational ties between leaders and the followers predict cheating at work (Trevino, 1986; Brass et al., 1998; Treviño et al., 2006; Cohn et al., 2014). The predominant part of this line of research has focused on the role of leadership in promoting or impeding cheating at work (Djawadi and Fahr, 2015; Paterson and Huang, 2018). Based on the behavioral integrity of the leaders as perceive by the followers (Kiersch and Byrne, 2015), leaders play a vital role in explaining and promoting desirable outcomes and preventing the outcomes that are not required (Leroy et al., 2012), thus, affecting cheating behavior of their subordinates (Cianci et al., 2014; Braun and Hornuf, 2018).

### Performance pressure and cheating behavior

Performance pressure is defined as the factors that increase the magnitude of performing well at workplaces (Baumeister, 1984). The employees who feel performance pressure at their workplaces believe that high performance is required at work and the efforts to perform high will be linked to their distal consequences and that their efforts to perform workplace tasks will be scrutinized in a high-stakes manner (Gutnick et al., 2012; Sheng and Fan, 2022). Meeting high demands will lead to enhanced standing of the individuals; however, failure to meet the workplace’s high demands may put him/her in danger at work. Researchers have found that the performance pressure is a threatening experience for the employees as it questions the current efforts of the employee concerning high performance demand (Sitkin et al., 2011), indicating the inadequacy of current performance for attaining the required demanded output (Zhang et al., 2017). The employees then try to elevate their efforts to meet the demanded output by stretching their capabilities, which at times are impossible to manage (Baumeister, 1984; Shalley and Perry-Smith, 2001).

In addition to performance pressure as a work demand, employees well understand that workplace efforts are linked with distal consequences (Gutnick et al., 2012), not meeting the high demands of the workplace may bring undesired negative outcomes. Employees sometimes engage in promoting and protecting self-interest by engaging in unethical practices and fabricating performance levels. In contemporary organizations, employees feel more pressure, employers are more demanding, they pressure their employees to elevate their performance ever increasing (DeZoort et al., 2006; Gutnick et al., 2012). Thereby, employees feel more pressure to be seen as performers at work by raising their performance level, otherwise, they have to face undesirable consequences, such as salary deductions, contract terminations, and/or some sort of punishments (Gutnick et al., 2012).

Researchers have also found that when employees perceive their work task based on performance goals, they are motivated to engage in unethical behaviors and they are also motivated to exaggerate their work performance (Schweitzer et al., 2004; Welsh and Ordóñez, 2014). Thereby, by putting pressure on employees to enhance their performance, the organizations might be inadvertently and actively promoting cheating behavior of their employees (Chen and Chen, 2021; Campos et al., 2022). The belongingness to a social work group also emerges as a predictor of performance pressure on employees, the threat of being excluded from a social work group makes anger a likely response, thereby promoting employees’ cheating behavior (Mitchell et al., 2018; Spoelma, 2021). Thereby, performance pressure threatens employees, elicits self- interests, and promotes cheating behavior of employees as a way to obtain undeserved benefits by fabricating performance level for addressing increasing performance demands. Because, by nature, the performance pressure threaten employees and produces paradoxical reactions (Baumeister, 2002), when employees experience performance pressure, their unethical intentions increases (Deng et al., 2022; Zhu et al., 2022).and their cheating behavior is enhanced (Mitchell et al., 2018; Spoelma, 2021).

### Leaders’ ambition and subordinates’ performance pressure

Ambition is a desire to achieve ends, especially, ends like success, power, and wealth (Hansson et al., 1983). Scholars have taken different theoretical perspectives to explain ambition (Judge and Kammeyer-Mueller, 2012), the central to all of these different theoretical perspectives is the aspirational nature of ambition- the motivational process that energizes individuals for the attainment of outcomes. Ambition theory (Schlesinger, 1966) explains the ambition of a person as the degree to which a person seems socially self-confident, leader-like, competitive, and energetic (Hogan et al., 2007). These persistent efforts to strive for success, attainment, and accomplishment (Judge and Kammeyer-Mueller, 2012) benefit organizations by enhancing a high level of commitment and performance (Steffens et al., 2018). Generally, it has been conceptualized as a psychological level phenomenon and treated as an individual-level trait (Hansson et al., 1983), however, ambition has also been affected by socioeconomic factors (Sewell et al., 2003). Recent research has found that ambition by definition, is a facet of conscientiousness or extroversion (Hogan et al., 2007; Judge and Kammeyer-Mueller, 2012). Ambition is an extroversion facet of an individual’s personality (Hogan et al., 2007), and the leaders with this feature (Digman, 1990) show more consistency in their effort to perform better along with their followers (Judge et al., 2002).

The tradition in psychological research is to explain ambition with respect to goals, plans, and accomplishments (Locke and Latham, 2002), however, ambition is more about attaining than achieving (Judge and Kammeyer-Mueller, 2012). Ambitious individuals with strive to achieve high targets in life, will put more efforts to achieve a higher level of education (Meyer, 1977), will have a satisfying career and social status, and receive better grades (Kim and Schneider, 2005). To achieve higher levels of financial rewards is also high for ambitious individuals, one of the core features of ambitious individuals is having a desire to achieve financial success, thus, with the achievement of personal wealth they signal that they have succeeded and attained success (Judge and Kammeyer-Mueller, 2012). A high level of occupational success is also a sign of attainment and attractiveness for ambitious individuals, therefore, individuals with high ambitions translate their intentions to perform into practice (Rhodes et al., 2005). Organizations also set ambitious goals to shorter duration of unemployment (Kanfer et al., 2001), more financial success (Nickerson et al., 2007), and highly creative achievement of their employees (Helson and Srivastava, 2002). Overall, research has established that ambitious individuals- due to their aspirational nature, put more effort into their activities and attain high targets in life (Judge and Kammeyer-Mueller, 2012).

Leaders’ role remained significant for performance pressure, employee’s actual performance and un-ethical behavior (Zhang et al., 2021, 2022; Liu et al., 2022). Ambitious leaders are needed by organizations (Steffens et al., 2018). They do whatever it takes to grow their business even at the cost of some necessary sacrifices (Gundry and Welsch, 2001). Ambitious leaders seize the opportunities to change things for the betterment (Balda and Mora, 2011), they achieve high goals (McClelland, 1975), which ultimately benefit organizations in the form of high employee commitment and better performance (Steffens et al., 2018). Followers of an ambitious leader tend to demonstrate high levels of achievements (Dilchert, 2007) or are more result-oriented (Winsborough and Sambath, 2013; Sieberer and Müller, 2017), to some extent, these studies support that with high ambitious leader followers have to take more ownership and pressure to achieve leaders’ goals. Thus, employees working with an ambitious leader must feel pressure to perform their routine tasks.

*H1*: There is a positive association between leaders’ ambition and subordinates’ performance pressure.

### The moderating role of leader identification

Social identity theory (Ashforth and Mael, 1989) has been used to explain employees’ self-identification with their organizations, groups, and with their leaders (Chen et al., 2015; Chughtai, 2016; Nason et al., 2018). The core premise in social identity theory is identifying and belongingness of an individual with others based on some attributes, characteristics, or attractions. The identification with the leader is a relational self-based on the characteristics they see in the leaders they use to define themselves (Wang and Rode, 2010). The identification with a leader works from two sides: follower recognize that he or she shares similar characteristics with the leader, and/or desire to change his or her self-concept so that his or her values and beliefs become similar to the leader (Pratt, 1998). Leader identification reflects the extent to which followers believe that their leader is self-defining (Kark et al., 2003); that is the perception of the followers’ oneness with the leader (Ashforth et al., 2016). In one of the few studies to investigate leader identification, researchers found that when followers have high identification with their leader, they exert more effort to meet the expectations of their leader for creativity (Wang and Rode, 2010). It appears that investigating how identification with a leader relates to a leader’s ambitions is essential for a proper understanding of the effects of leaders’ ambition. Extending our previous discussion on the topic, here we suggest that followers’ perception of identification with their leader integrate with their leaders’ ambition for the magnitude of performance pressure.

Researchers have stated that followers’ identification with their leader leads individuals to experience the minimal distinction between themselves and their leader (Andersen and Chen, 2002), enhancing commitment and consideration toward their leader (Sluss and Ashforth, 2007). Followers with high identification with their leaders perceive that acting for the benefits and need of their leader is acting for their benefits and needs; they become more sensitive toward the behavior of their leaders, success of their leaders, expectations of their leaders, and needs of their leader (Berscheid and Reis, 1998; Van Knippenberg et al., 2004). Accordingly, we posit that followers’ identification with their leader will play a vital role in explaining and determining whether leaders’ ambition is associated with performance pressure that employees feel at workplaces. Research has proposed that followers’ identification with their leaders must augment with leaders’ behavior for a higher level of influence on the followers’ performance (Van Knippenberg et al., 2004; Wang and Rode, 2010).

*H2*: The positive association between leaders’ ambition and subordinates’ performance pressure is strengthened when subordinates also have high levels of leader identification.

### The mediating role of performance pressure

Previous studies on leaders’ ambition suggest that the motivational process of aspiration energizes individuals to evaluate their own competence and set progressive goals to achieve desired results with high growth strategies (Gundry and Welsch, 2001; Judge et al., 2002; Hogan et al., 2007). Leaders are considered to be a continuous source of pressure for the employees (Kamran et al., 2022; Sheng and Fan, 2022). Ambitious leaders require high performance from their subordinates, thereby employees may feel more performance pressure. Researchers found that ambitious leaders set high performance goals with higher growth strategies to achieve higher goals (Winsborough and Sambath, 2013). Consequently, increasing the motivational basis for professional and organizational commitment to achieve high performance goals (Desrochers and Dahir, 2000), thereby, enhancing performance pressure on the followers (Roberts et al., 2007; Winsborough and Sambath, 2013).

We contend that performance pressure may mediate the relationship between leaders’ ambition and subordinates’ cheating behavior. As noted above, more ambition of the leaders is associated with high performance pressure of the followers because high ambition and expectations of the leaders are translated into followers’ high attainment, accomplishments, and need for generalized striving for success (Judge and Kammeyer-Mueller, 2012) and because it may create an impression of the subordinates’ high achievement among their supervisors (Locke and Latham, 2002). In addition, there is accumulating evidence that subordinates perform better when they have positive workplace associations (Adeel et al., 2019b), when they define themselves as identical to their leaders (Wang and Rode, 2010), and when their leaders are high in ambitions (Steffens et al., 2018).

Although ambition is often not generally viewed as having negative consequences, it is still unclear whether it has vice or virtue consequences (Pettigrove, 2007; Judge and Kammeyer-Mueller, 2012). In particular, some researchers have argued that the leaders’ ambition has a positive effect on the behavior of their subordinates (Locke and Latham, 2002; Judge and Kammeyer-Mueller, 2012) when driven by identification of the subordinates with their leaders (Wang and Rode, 2010). Hence, here we believe that identification with leaders may influence leaders’ ambition on the cheating behavior of employees in the same way as it influences the effects of leaders’ ambition on performance pressure. Consequently, we contend that identifying with one’s leader may interrupt the performance pressure as triggered by the leader’s ambition. Therefore, we predict a mediated moderation as depicted in the hypothesis below.

*H3*: Performance pressure mediates the interactive effects of leaders’ ambition and subordinates’ leader identification on subordinates’ cheating behavior.

**Figure 1:**
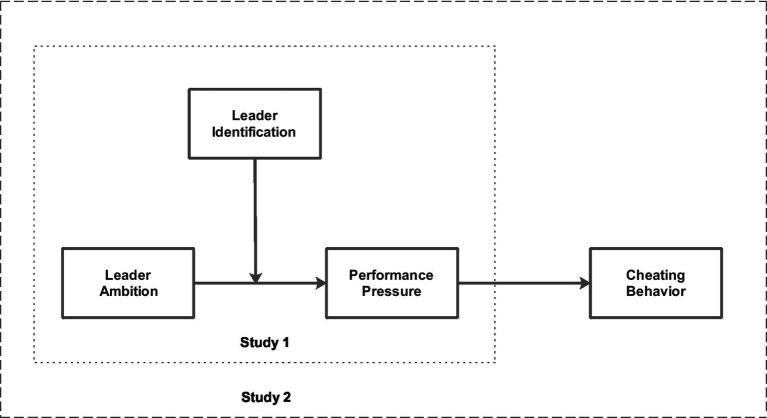
Research model.

Data for the hypothesized relationships was collected in two separate studies. Since in study 1 we could not collect data for employees’ cheating behavior (police officers), we only tested hypotheses 1 and 2 in this study. In study 2, we collected data for all of our Islamic banking professionals’ variables and examined all the hypotheses. The key theoretical relationships are presented in Figure 1. Previous researchers have used non-probability sampling by recruiting a variety of adequate participants (Erdogan et al., 2015; Adeel et al., 2022b), therefore, following previous research we have also used convenience sampling.

## Study 1

### Sample and data collection

Data for this study was sampled from a police academy in Punjab province. The officers were on the job training for intermediate and upper-class courses with 16 weeks duration. Studying performance pressure and its relationship with leader ambition and leader identification on a sample of police officers appears practically interesting due to the high stressful performance demand of this job (Romosiou et al., 2018; Zempi, 2018) and the relevance of the research question with the context of this study. In February 2019, an Initial email was sent to all of the 437 officers who were part of the training center. Three hundred and thirty one showed their willingness *via* return email, we then sent a questionnaire to those willing to participate in this study. The initial response was received from 218 officers and all of their 6 training managers. After deleting data with missing values and mismatched with instructors’ response, our final sample yielded 196 from officers and 6 from their relevant instructors with a response rate of 59 and 100%, respectively. This final sample included officers from (criminal investigation, security police, and police command). In our final qualified sample, over half of the respondents (51%) with; the average age of participants was 22.81 years.

### Measures

Leaders’ Ambition: We measured leaders’ ambition with five items ambition scale (Duckworth et al., 2007) adapted by (Rodriguez et al., 2013). Course instructors responded on this -reporting measure of leaders’ ambition with a five-point Likert scale ranging from 1 = not at all like me to 5 = very much like me. Scale items are “I aim to be the best in the world at what I do,” “I am ambitious,” “Achieving something of lasting importance is the highest goal in my life,” “I think achievement is overrated,” and “I am driven to succeed” (*α* = 0.86).

Performance Pressure: We measured performance pressure (Mitchell et al., 2018) with four items five-point Likert type scale ranging from 1 = Strongly disagree to 5 = Strongly agree. Officer provided their response for this self-reporting measure. Sample scale items are “The pressures for performance in my workplace are high” and “If I do not produce at high levels, my job will be at risk” (*α* = 0.92).

Leader Identification: We measured leader identification (Kark et al., 2003; Wang and Howell, 2012) with six items five-point Likert type scale ranging from 1 = Strongly disagree to 5 = Strongly agree. Officers provided this response on this self-reporting measure. The sample item is “My supervisors’ successes are my successes” (*α* = 0.78).

Control Variables: Research has shown that demographic variables influence the perception of pressure (Kish-Gephart et al., 2010). Therefore, we controlled for subordinates’ gender, age, and organizational tenure. Researchers have argued that moral identity may also affect the performance pressure (Chen and Chen, 2021) and moral development and behaviors (Kohlberg and Power, 1981; Aquino and Reed, 2002). Therefore, we controlled for moral identity in both of our studies with five items five-point likert type scale (Aquino and Reed, 2002).

### Descriptive statistics

Mean, standard deviation, and correlations among the study variables are depicted in Study 1-Table 1.

**Table 1:** Study 1 - means, standard deviation, and correlation among study variables.

Variable	Mean	SD	1	2	3	4	5
1. Gender	0.49	0.50					
2. Age	22.81	1.54	−0.048				
3. Moral identity	3.04	0.63	0.002	0.059			
4. Leader ambition	3.46	0.74	0.047	−0.005	0.087		
5. Leader identification	3.87	0.70	0.155*	−0.060	−0.053	0.073	
6. Performance pressure	3.62	0.84	0.141*	0.111	0.104	0.296**	0.176*

Observations = 196. Clusters = 6. Gender was coded as 0 = Female, 1 = Male. Education was coded as 1 = College Graduate, 2 = Bachelor Degree, 3 = Master Degree. S.E. = standard error. *p < 0.10; **p < 0.05; ***p < 0.01.

### Results

Due to the nested nature of the study sample, linear regression for this study could underestimate standard error. Therefore, on recommendations of researchers for nested data (Scherbaum and Ferreter, 2009), we used random coefficient modeling for a single level of analyzes with Mplus 7.3 (Muthén and Muthén, 2010). Although the technique eliminates chances of standard error underestimation and potential interdependence among study variables, the output produced with this technique cannot be used for model fit indicators regularly. Therefore, for chi-squared different testing, we performed the Satorra-Bentler difference test using the log-likelihood method with the scaling correction factor (Muthén and Muthén, 2010). Main study variables, control variables, and interaction term also grand mean-centered before any analyzes of this study.

**Figure 2:**
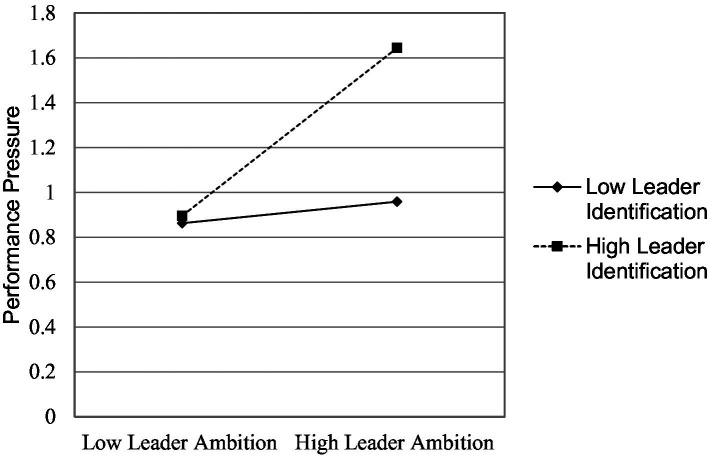
Study 1: interaction of leader ambition and leader identification.

Random coefficient regression analyzes are depicted in Study 1-Table 2. For this study we were interested to know the effects leader ambition could have on performance pressure, therefore, our core hypothesis was about the relationship between leader ambition and performance pressure. Results in Study 1-Table 2 model 2 shows that leader ambition is a positive predictor of performance pressure (*β* = 0.325, *p* ≤ 0.05). The interaction term of leader ambition and leader identification in the Study 1-Table 2 model 3, also emerged as a positive predictor of performance (*β* = 0.283, *p* ≤ 0.001). Study 1-Figure 2 depicts interaction findings; the results of this interaction show that performance pressure is higher when both leader ambition and leader identification are high. High leader ambition is likely to produce a higher level of performance pressure when subordinates also had a high level of leader identification. The results of this study supported both hypotheses of this study.

**Table 2:** Study 1- regression analyzes.

Predictor	Model 1 performance pressure		Model 2 performance pressure		Model 3 performance pressure	
	Estimate	SE	Estimate	SE	Estimate	SE
						
Gender	0.246***	0.090	0.224	0.087	0.165	0.099
Age	0.061	0.035	0.063	0.033	0.082**	0.033
Moral identity	0.129	0.160	0.096	0.115	0.093	0.098
Leader ambition			0.325**	0.142	−0.806	0.467
Leader identification					−0.752**	0.308
Leader ambition X leader identification					0.283***	0.097
Δ*χ*^2^(Δdf)	3.20(2)		10.64(3)**		24.73(5)***	
Δ*R*^2^	0.037		0.60		0.34	

Observations = 196. Clusters = 6. Gender was coded as 0 = Female, 1 = Male. Education was coded as 1 = College Graduate, 2 = Bachelor Degree, 3 = Master Degree. S.E. = standard error. Δχ^2^ refers to Satorra–Bentler scaled chi-square difference test: Muthén and Muthén (1998). Δdf is change in degrees of freedom. *R*2 is the proportional reduction in error variance (Snijders and Bosker, 2011). *p < 0.10; **p < 0.05; ***p < 0.01.

## Study 2

### Sample and data collection

To increase the generalizability and validity of proposed relationships, we conducted a second study, using data collected from Islamic banking professionals. Since, in study 1, we could not collect data for employees’ cheating behavior, we only tested hypotheses 1 and 2. In study 2, we collected data for leader ambition, leader identification, performance pressure, and cheating behavior. Thus, we examined all the hypotheses, including mediated moderation in study 2.

The sample of study 2 included 374 responses from their respective 37 managers from an Islamic bank operating in Pakistan. The claim that Islamic banking has an ethical foundation is based on the Islamic principles of equity, cooperation, and social justice (Ismaeel and Blaim, 2012; Mansour et al., 2015). Therefore, Islamic banking operations and management are considered best suited for this study’s purpose and scope.

We discussed the purpose, scope, and relevance of the study with the management of the bank and obtained approval for data collection. For this study, we used two sources of data collected from managers and their respective subordinates by dividing the data collection process into three points in time. HR department sent a questionnaire to 758 subordinates and their respective 58 supervisors at time 1. The response was received from 621 subordinates at time 1, 590 subordinates at time 2, and 394 subordinates at time 3. After deleting records with missing values and mismatched data with managers’ responses, our final sample yielded a subordinates’ response of 374 and the managers’ response of 37 (49 and 65% respectively). Subordinates provided their response for demographic variables, organizational experience, moral identity, organizational identity, and leader identification at time 1; they also provided their response for performance pressure and cheating behavior at times 2 and 3. Managers provided their response for leader ambition at time 1 only. Thus, subordinates provided data for demographic variables, organizational experience, moral identity, organizational identity, leader identification, performance pressure, and cheating behavior, and managers provided their response for leader ambition. In this study’s final qualified sample, 66% were males and 34% were females; the average age of the subordinates was 39.70 years; an average of organizational experience was 7.246 years.

### Measures

*Leaders’ Ambition:* We measured leaders’ ambition using five items ambition scale (Duckworth et al., 2007) adapted by (Rodriguez et al., 2013) as used in study 1 (*α* = 0.95). *Performance Pressure:* We used a 4-item scale to measure performance pressure (Mitchell et al., 2018) as used in study 1 (*α* = 0.93). *Leader Identification:* We measured leader identification with the same six items scale (Kark et al., 2003; Wang and Howell, 2012) as used in study 1 (*α* = 0.85).

Control Variables: Similar to study 1, we also controlled for subordinates’ gender, age, experience with current organization, and moral identity, which may affect cheating behavior (Kohlberg and Power, 1981; Aquino and Reed, 2002; Kish-Gephart et al., 2010). Research has shown that organizational identification influences organizations’ unethical behavior (Chen et al., 2016). Consequently, we controlled for organizational identification with six items five-point Likert type scale (Mael and Ashforth, 1992). Scale items range from 1 = Strongly disagree to 5 = Strongly agree. Sample scale items are “When someone criticizes my organization, it feels like a personal insult” and “I am very interested in what others think about my organization” (*α* = 0.83).

### Data analysis

#### Results

Study 2-Table 3 reports descriptive statistics and zero-order correlation among the study variables.

**Table 3:** Study 2- means, standard deviation, and correlation among study variables.

Variable	Mean	SD	1	2	3	4	5	6	7	8
1. Gender	0.66	0.47								
2. Age	39.70	5.39	0.100							
3. Experience^a^	7.246	2.56	0.115*	0.171**						
4. Moral identity	3.19	0.28	−0.065	0.008	0.091					
5.Organizational identification	3.81	0.55	0.093	−0.090	−0.111*	−0.011				
6. Leader ambition	3.33	0.88	0.056	−0.018	0.001	0.132*	−0.055			
7. Leader identification	3.84	1.03	0.071	0.053	0.007	−0.020	−0.066	0.064		
8. Performance pressure	3.55	0.60	0.017	−0.085	0.005	−0.001	−0.074	0.213**	−0.188**	
9. Cheating behavior	3.75	0.75	0.083	0.016	0.010	0.109*	0.035	0.167**	−0.145**	0.258**

Observations = 374. Clusters = 37. Gender was coded as 0 = Female, 1 = Male. Education was coded as 1 = College graduate, 2 = Bachelor degree, 3 = Master degree, 4 = Doctoral degree. Professional experience was measured in years. S.E. = Standard error. ^a^Experience with current organization. *p < 0.10; **p < 0.05; ***p < 0.01.

#### Confirmatory factor analyzes

In order to evaluate discriminant validity of study variables, we conducted a CFA using Mplus 7.3 (Muthén and Muthén, 2010). Item level indicators were modeled which provided a good fit to the data *χ*^2^ = 10824.024 (703), *p < 0*.01; CFI = 0.95; TLI = 0.94; RMSEA = 0.07 as compared to all other alternative constrained models illustrated in Table 4. These CFA results demonstrated that the four-factor model had satisfactory discriminant validity.

**Table 4:** Study 2- comparison of measurement models.

Models	Factors	*χ* ^2^	df	CFI	TLI	RMSEA
Baseline model	Four factors	10824.024	703	0.95	0.94	0.07
Model 1	Three factors: leader ambition and performance pressure combined into one factor	12943.312	706	0.88	0.86	0.09
Model 2	Three factors: leader ambition and leader identification combined into one factor	13421.065	706	0.87	0.86	0.09
Model 3	Two factors: leader ambition, performance pressure, and leader identification combined into one factor	11663.532	708	0.90	0.89	0.08
Model 4	One factor: all variables combined into one factor	11872.023	709	0.91	0.91	0.08

## Results

Mplus 7.3 (Muthén and Muthén, 2010) was used to test hypotheses in this study. We used random coefficient regression analyzes operated at a single level as used in study 1. Data collected from bankers was nested in nature; therefore, the use of OLS regression could underestimate standard error (Scherbaum and Ferreter, 2009). Additionally, due to the potential problem of interdependence among study variables, we used random coefficient modeling at a single level of analysis for all these study models. The output produced with random coefficient modeling cannot be used in a regular way for model fit indicators (Muthén and Muthén, 2010). Therefore, we performed the Satorra-Bentler difference test using the log-likelihood method for null and alternate models with scaling correction factors. Same methods of analysis have already been used by researchers with data of similar characteristic (Adeel et al., 2022a,b). We also grand mean centered all the study variables, including interaction terms (Aiken et al., 1991; Hofmann and Gavin, 1998). Random coefficient analyzes results are depicted in Study 2-Table 5. Bootstrapping cannot be used for indirect effects with random coefficient analyzes (Muthén and Muthén, 2010). Therefore, for random coefficient mediated moderation, we followed the three-step procedure of mediated moderation (Baron and Kenny, 1986; Muller et al., 2005).

**Table 5:** Study 2- summary of hierarchical random coefficient regression analysis results.

Predictor	Model1 cheating behavior		Model2 performance pressure		Model3 cheating behavior	
	Estimate	SE	Estimate	SE	Estimate	SE
Control variables						
Gender	0.024	0.061	0.121	0.063	0.127	0.063
Age	−0.010	0.007	0.002	0.010	0.005	0.008
Experience with current organization	−0.002	0.012	0.003	0.011	−0.003	0.011
Moral identity	−0.059	0.135	0.254	0.181	0.262	0.167
Organizational identification	−0.080	0.068	0.054	0.092	0.065	0.084
Independent variable						
Leader ambition	0.143**	0.058	0.130**	0.063	0.097**	0.048
Moderator						
Leader identification					−0.084	0.049
Mediator						
Performance pressure					0.273***	0.090
Δ*χ*^2^ (Δdf)	726.78 (4)***		711.36 (4)***		1122.66 (7)***	
Δ*R*^2^	0.088		0.272		0.117	
Independent variable						
Leader ambition	−0.307	0.203	−0.247**	0.107	−0.234	0.190
Moderator						
Leader identification	−0.494***	0.183	−0.458***	0.114	−1.026	0.477
Interactive effect						
Leader ambition X leader identification	0.181**	0.089	0.1261***	0.044	0.083	0.049
Δ*χ*^2^ (Δdf)	594.12 (6)***		655.84 (6)***		15.08 (8)*	
Δ*R*^2^	0.147		0.303		0.058	
Mediator						
Performance pressure					0.378***	0.106
Interactive effect						
Performance pressure X leader identification					−0.016	0.013
Δ*χ*^2^ (Δdf)					853.63(5)***	
Δ*R*^2^					0.088	

Observations = 374. Clusters = 37. Gender was coded as 0 = Female, 1 = Male. Education was coded as 1 = College graduate, 2 = Bachelor degree, 3 = Master degree, 4 = Doctoral degree. Professional experience was measured in years. S.E. = standard error. *p < 0.10; **p < 0.05; ***p < 0.01. Δχ^2^ refers to Satorra–Bentler scaled chi-square difference test: Muthén and Muthén (1998). Δdf is change in degrees of freedom. *R*^2^ is the proportional reduction in error variance (Snijders and Bosker, 2011).

**Figure 3:**
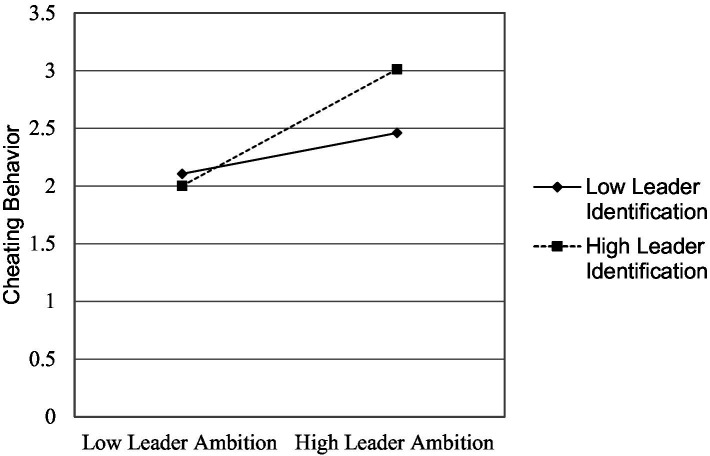
Study 2: interaction of leader ambition and leader identification.

**Figure 4:**
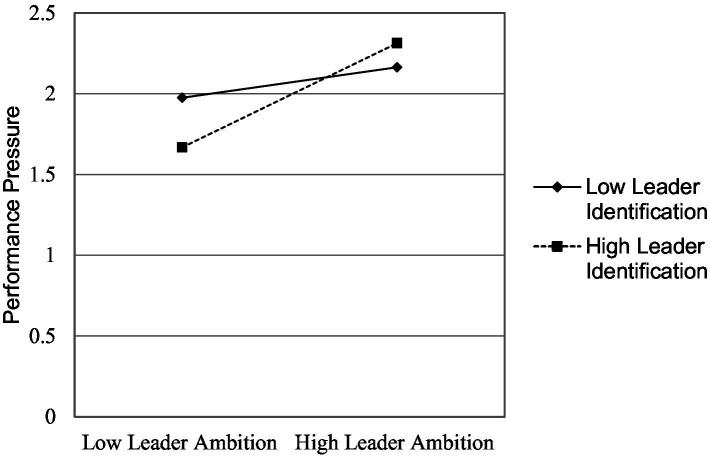
Study 2: interaction of leader ambition and leader identification.

As in study 1, we also examined the direct effect of leaders’ ambition on performance pressure and the interaction of leaders’ ambition and leader identification on performance pressure. We used subordinates’ gender, age, experience with current organization, moral identity, and organizational identification as controls; leaders’ ambition as the independent variable; performance pressure as mediator; and leader identification as a moderator in this study. Study 2-Table 5 (model 1 and 2), leaders’ ambition is a positive predictor of cheating behavior (*β* = 0.143, *p* < 0.05, Δ *R^2^* = 0.088) and performance pressure, respectively, (*β* = 0.130, *p* < 0.05, Δ*R^2^* = 0.272). As shown in Study 2-Table 5 (model 1), the interaction term of leaders’ ambition and leader identification also emerged as a positive predictor of cheating behavior (*β* = 0.181, *p* < 0.05, Δ*R^2^* = 0.147). With these results, we fulfilled the first requirement of the mediated moderation model. The interaction results are depicted in Study 2-Figure 3. The results demonstrate that cheating behavior is higher when both leaders’ ambition and leader identification are high. Study 2-Table 5 (model 2), the interaction term of leaders’ ambition and leader identification also emerged as a positive predictor of performance pressure (*β* = 0.126, *p* < 0.01, Δ*R^2^* = 0.303); fulfilling the second requirement of the mediated moderation model. The interaction results are shown in study 2-Figure 4. The results show that performance pressure is higher when both leaders’ ambition and leader identification are high.

Finally, in study 2-Table 5 (model 3), the results revealed that performance pressure has a significant mediating effect on cheating behavior in presence of all control variables, leaders’ ambition, leader identification, the interaction of leaders’ ambition and leader identification, and interaction of performance pressure and leader identification (*β* = 0.378, *p* < 0.01, Δ*R^2^* = 0.088), however, the coefficient of the interaction term of leaders’ ambition and leader identification became insignificant (*β* = 0.083, *p* ˃ 0.10, Δ*R^2^* = 0.058); fulfilling the final requirement of the mediated moderation model. The results indicate that performance pressure fully mediates the interaction effect of leaders’ ambition and leader identification on cheating behavior of employees. With the results of this study, we provided support to all of the hypotheses of this research.

## Discussion

We found that leaders’ ambition is more strongly related to subordinates’ performance pressure and subordinates’ cheating behavior when leader identification is high. These findings suggest that leaders themselves can be sources of performance pressure and cheating behavior of employees at organizations. Subordinates with high leader identification take more pressure and are likely to demonstrate more cheating when working with ambitious leaders. Indicating that a high level of loyalty to the leader is dangerous for the organizations. Thus, the results of this research have extended leaders’ ambition literature by demonstrating the role and importance of subordinates’ leader identification for subordinates’ performance pressure and subordinates’ cheating behavior.

### Theoretical implications

This research offers some distinct contributions to the literature. This research’s primary contribution lies in answering the fundamental question that what leaders and organizations are unintentionally doing, may motivate employees for cheating. Researchers have long been emphasizing the need for organizations that motivate employees to behave unethically (Moore and Gino, 2013; Mitchell et al., 2018). We theorized that organizations’ cheating behavior occurs when leaders heighten their achievement of goals by energizing employees to use their full potential for the betterment of the organizations, which they do when they pressure their subordinates to raise performance. Prior research has explored the role of high performance pressure on employees’ cheating behavior (Mitchell et al., 2018). This research line has shown that in order to overstate productivity, organizations unintentionally enhance employees’ self-interested motives and need for self-protection that elicits employees’ cheating behavior through enhanced performance pressure. We enhanced this research line, further unpacking the reasons when and how performance demands as fueled by leaders’ ambitions promotes the cheating behavior of employees. We argue that leaders’ high ambitions of achievements for their organizations enhance their demand from the subordinates for high performances- a subjective experience of the employees to raise their efforts for high performance linked with distal consequences promotes employee cheating behavior. Thus, leaders’ ambition becomes a threatening cause and elicits employee cheating behavior, which is internalized through the subjective experience of pressure to perform high.

We extend the literature on leadership by investigating how leaders’ ambitions are translated into performance pressure and cheating behavior of employees. The results of this research provide more understanding of the consequences of leaders’ ambitions. The presumed consequences of high ambitious leaders are beneficial for the organizations, they energize their followers (Steffens et al., 2018) for the achievement of goals (McClelland, 1975) and for the benefits of their organizations (Balda and Mora, 2011; Saleem et al., 2020). We add to this line of research by explaining the potential negative side of leaders’ ambitions. This addition to the previous research efforts is important because leaders focus on the employees for the achievement of goals in the work context. Additionally, this research also explained that subordinates with high leader identification are expected to take more pressure to perform, thereby enhancing their cheating intentions. This research highlighted the process that explains why and how leaders’ ambitions are translated into employees’ cheating behaviors.

We shift the focal point away from the analysis of leaders and followers separately, a much-needed shift (Shamir, 2007; Bastardoz and Van Vugt, 2019), previous research has rarely investigated leaders and followers in a single study (Day, 2014; Bastardoz and Van Vugt, 2019). The main reason remained; followers are considered the default in leadership research ignoring the fact that they are not just a monolithic group (Carsten et al., 2010) but individuals with different emotions, behaviors, abilities, aptitude, and motivation that are affected by leaders (Bastardoz and Van Vugt, 2019). Therefore, for a deeper understanding of leaders and followers, and the behavior of followers triggered by leaders, a shift of conventional investigation lens was needed. Our final contribution is concerned with the integration of ambition theory with social identity theory, in our point of view, provides a more comprehensive and integrative model to understand leaders’ deeds that are translated into followers’ behaviors, more specifically, the positive deeds of leaders that are promoting negative behaviors of followers.

### Managerial implications

This study’s practical implication is that leaders’ positive desire in general and ambition, in particular, may not be necessarily associated with subordinates’ positive behaviors. Our results revealed that internalized with performance pressure, the leaders’ ambitions are associated with subordinates’ cheating behavior. The high ambitions of leaders are associated with high cheating behavior and high performance pressure for those subordinates who also had high identification with the leaders. The results help us understand what might be enhancing performance pressure and ultimately cheating behavior of subordinates, therefore, it is critically important for the organizations to not only have ambitious leaders but also subordinates of an ambitious leader with low leader identification so that the intentions of cheating could be minimized. Customized training programs for the development of organizational identification in employees may help prevent the elicitation of cheating behaviors by shifting the focus of the employees from identification with their leader to identification with their organization.

It is known that subordinates’ identification with their leader as a relational bias affect the oneness perceptions of subordinates with their leader (Ashforth et al., 2016) where meeting leaders’ expectations become the prime interest of the subordinates (Wang and Rode, 2010) ignoring the expectations of their organization (Chen et al., 2015; Chughtai, 2016; Nason et al., 2018). If a subordinate, who genuinely wants to enhance work performance for his/her organization, mistakenly attributes his/her identification with the leader as identification with his/her organizations, is likely to be stirred toward more pressure to perform and cheating. Employee development programs designed to develop and enhance ethics may also help reduce the cheating intentions of employees. Discussion forums at the organization’s web portal, operated by high officials, could be used to shift employees’ identification from their leaders to identification with their organization. These discussion forums could also be used for employees’ ethical development and for communicating proper performance measures as needed by their organization. These transparency measures would increase the organization’s direct communication to the employees, organization’s direct communication to the employees, leaving fewer chances for leaders to manipulate situations for high performance demands, ultimately promoting cheating behaviors.

### Limitations and future research directions

Like any study, this research is also not free from limitations. Although we have strong theoretical reason to expect that leader ambition would precede performance pressure and/or cheating behavior and not vice versa, the research design of this research does not allow us to test the temporal order of the study variables, and the conditions under which cheating behavior would precede performance pressure and/or leader ambition. The theoretical reason we expect that leader ambition would precede performance pressure and/or cheating behavior and not vice versa is that leaders’ ambition literature predominantly shown that the ambitious leaders energize followers (Steffens et al., 2018) to use their full potential (Hobfoll, 2011) for the achievement of goals (McClelland, 1975).

Empirically, however, we could not tease apart the causality of the proposed relationships among the study variables. One possible reason for this limitation would be the age and the professional experiences, even though, the supervisors in our research had legitimate positions in organizations, they had an almost similar level of professional experience [7.24 years for the subordinates and 8.37 years for the supervisors (study 2)] and age (22.81 and 39.7 years for the subordinates for study 1 and study 2 respectively; 23.37 and 41.54 years for the supervisors for study 1 and study 2 respectively). A possible different interpretation of our results could be that when subordinates take pressure on their assigned tasks, they are energized for high ambitions. However, our findings at least provided some evidence that treating leader ambitions as an antecedent, rather than a consequence, of cheating behavior is consistent with theory as well as the sample characteristics we studied. Still, future research should seek to further explore the directionality issue, in a longitudinal experimental design.

Another limitation of this study lies in the context of this research. We collected data from the police officers in study 1 and from the Islamic banking professional in study 2, although both of these departments are expected to be ethical. However, we found positive results for performance pressure and cheating in an ambitious leader. However, it is still unclear whether the study results would generalize to disparate occupations, cultures, and at different organizational levels. Although the police department and banking profession are important employers worldwide, further research should focus on disparate occupations so that the results would be generalized. Thus, we recommend, future research should explore and operationalize the relationships among leaders’ ambitions, leader identification, performance pressure, and cheating behavior in industries other than banking and police departments where data collected from the employees working at different hierarchical levels.

## Data availability statement

The raw data supporting the conclusions of this article will be made available by the authors, without undue reservation.

## Ethics statement

The studies involving human participants were reviewed and approved by The University of Chenab. The patients/participants provided their written informed consent to participate in this study.

## Author contributions

All authors listed have made a substantial, direct, and intellectual contribution to the work and approved it for publication.

## Conflict of interest

The authors declare that the research was conducted in the absence of any commercial or financial relationships that could be construed as a potential conflict of interest.

## Publisher’s note

All claims expressed in this article are solely those of the authors and do not necessarily represent those of their affiliated organizations, or those of the publisher, the editors and the reviewers. Any product that may be evaluated in this article, or claim that may be made by its manufacturer, is not guaranteed or endorsed by the publisher.
